# Patients’ Self-Report and Handwriting Performance Features as Indicators for Suspected Mild Cognitive Impairment in Parkinson’s Disease

**DOI:** 10.3390/s22020569

**Published:** 2022-01-12

**Authors:** Sara Rosenblum, Sonya Meyer, Ariella Richardson, Sharon Hassin-Baer

**Affiliations:** 1 The Laboratory of Complex Human Activity and Participation (CHAP), Department of Occupational Therapy, Faculty of Social Welfare & Health Sciences, University of Haifa, Haifa 3498838, Israel; 2 Department of Occupational Therapy, Ariel University, Ariel 4077603, Israel; sonyam@ariel.ac.il; 3 Department of Industrial Engineering, Jerusalem College of Technology, Jerusalem 9372115, Israel; richards@jct.ac.il; 4 Movement Disorders Institute, Sheba Medical Center, Ramat Gan 5262000, Israel; sharon.hassin@sheba.health.gov.il; 5 Department of Neurology, Sheba Medical Center, Ramat Gan 5262000, Israel; 6 Sackler Faculty of Medicine, Tel Aviv University, Tel Aviv 6997801, Israel

**Keywords:** assessment, activities of daily living, functional cognition, Parkinson’s disease

## Abstract

Early identification of mild cognitive impairment (MCI) in Parkinson’s disease (PD) patients can lessen emotional and physical complications. In this study, a cognitive functional (CF) feature using cognitive and daily living items of the Unified Parkinson’s Disease Rating Scale served to define PD patients as suspected or not for MCI. The study aimed to compare objective handwriting performance measures with the perceived general functional abilities (PGF) of both groups, analyze correlations between handwriting performance measures and PGF for each group, and find out whether participants’ general functional abilities, depression levels, and digitized handwriting measures predicted this CF feature. Seventy-eight participants diagnosed with PD by a neurologist (25 suspected for MCI based on the CF feature) completed the PGF as part of the Daily Living Questionnaire and wrote on a digitizer-affixed paper in the Computerized Penmanship Handwriting Evaluation Test. Results indicated significant group differences in PGF scores and handwriting stroke width, and significant medium correlations between PGF score, pen-stroke width, and the CF feature. Regression analyses indicated that PGF scores and mean stroke width accounted for 28% of the CF feature variance above age. Nuances of perceived daily functional abilities validated by objective measures may contribute to the early identification of suspected PD-MCI.

## 1. Introduction

The reported frequency rate of mild cognitive impairment (MCI) among patients with Parkinson’s disease (PD), ranging from 20% to 50% [1,2], reflects the difficulty in defining and identifying PD-MCI. Early recognition of MCI is vital for both patients and their caregivers, enabling prompt intervention to lessen further emotional and physical health complications and improve patients’ life quality [3,4].

A central challenge lies in how to capture “the gradual cognitive decline, which does not significantly interfere with patients’ functional independence as subjectively reported by the patient and/or informant”, as determined by PD Brain Bank criteria [5]. These criteria lead to the need to assess functional cognitive decline and its possible consequences as reported by the patient. Functional cognition means the cognitive ability to perform daily tasks while incorporating varied cognitive domains. These motor/sensory skill domains support action and performance patterns, such as habits, routines, and roles, rather than specific cognitive skills, such as inhibition, attention, or memory, in isolation [6]. Functional cognition decline may be tied to negative emotional response, less efficiency and participation in activities of daily living (ADL) and life domains, and diminished life quality and depression [7,8]. Thus, we measured depression by the Beck Depression Inventory (BDI) test [9].

In a previous study, we presented the inadequate PD-MCI identification achieved by cognitive and neuropsychological tests due to the discorrelation found between test scores and individuals’ reports about their daily functional cognitive abilities [3]. We had emphasized the discrepancy between the results of the cognitive battery offered by the Movement Disorder Society (MDS) and the individuals’ self-reports of changes in their daily functioning related to cognitive decline. That study supported previous literature addressing the problem of making conclusions about human functional cognitive abilities from neuropsychological tests that evaluate each cognitive domain separately. Instead, this literature suggested the need for questionnaires/tests that relate to real-life functional scenarios that require integrating varied cognitive domains for efficient daily function [10,11].

We next presented the cognitive functional (CF) feature, which we created based on patients’ reports of certain items in the MDS Unified Parkinson’s Disease Rating Scale (UPDRS) [3,12]. The CF feature is calculated as the mean score of seven MDS-UPDRS-derived items: the cognitive impairment Item 1.1 from the UPDRS nonmotor experience of the daily living subscale and Items 2.1 speech, 2.4 eating, 2.5 dressing, 2.6 hygiene, 2.7 handwriting, and 2.8 doing hobbies from the UPDRS motor experience of the daily living subscale. As the CF feature has not been approved yet, we suggest the term suspected of PD-MCI based on results of these two prior studies (one in publication [3] and one in draft [4]) and participants’ reports of their real-life confrontations that are herein validated by objective measures. We assume the changes participants feel in their daily functioning are still too subtle for instruments such as the Montreal Cognitive Assessment (MoCA) to detect but significant enough to affect their functional capabilities.

In the second study, we found that PD patients defined as having suspected PD-MCI based on their self-reported CF feature exhibited significantly lower CF abilities than did PD patients without suspected MCI [4]. Furthermore, specific items of the self-reported Daily Living Questionnaire (DLQ) [13] and Time Organization and Participation Scale (TOPS) [14] accounted for up to 35% of the variance in the CF feature [4].

The results indicated that PD patients with suspected MCI feel something has changed in their CF abilities, as reflected in the amount of cognitive effort they need to invest in their daily activities and in their ability to organize time [4]. Their CF decline, emotional status, activity performance features, and motivation were related [15]. These relationships raise the question of how PD patients with suspected MCI would report on their general functional ability, satisfaction from their daily activities, and the changes in their roles compared to patients not suspected for MCI. It is further intriguing to consider whether the declined functional ability of patients suspected for MCI would manifest through objective measures of their actual activity performance, such as in their handwriting.

Handwriting is a complex human activity taught in childhood and a daily activity that adults perform in various settings. In a handwriting task, people operate and integrate lower-level perceptual–motor and higher-level cognitive processes [16]. Abnormal handwriting changes and specifically micrographia (abnormally small letter size) have been reported among PD patients [17]. Previous findings also indicated that handwriting measures potentially differentiate between PD and healthy subjects [18,19,20,21], and identify various stages of PD and both medication and intervention effects [22,23]. Thus, measures of handwriting also may support PD-MCI identification.

Past studies have indicated that using a computerized handwriting evaluation system, such as ComPET [24], to collect handwriting data enables an efficient and objective method to evaluate functional handwriting performance. Applying dynamic handwriting analysis in neurodegenerative disease assessment focused on PD also has been reviewed in the literature [25]. Specifically, digitized spiral drawing has been used to differentiate between PD and healthy controls [26,27]. Tasks in which sensing user handwriting with a digitized tablet was used include demonstrating performance change in patients with mild major depressive disorder (MDD) [28], differentiating between PD patients and healthy controls [17,29,30], and reflecting executive functions among patients with developmental coordination disorders [31,32]. In a previous PD study [17], 20 people with PD and 20 healthy people wrote their full names and copied an address. The variables that made the greatest contribution to group membership in that study—and thus were implanted in the current study—were mean width, duration, and height in both tasks. Based on these functions, we were able to classify 97.5% of participants as with or without PD. However, the distinction between PD with and without PD-MCI is not trivial. Therefore, in the current study, we used a handwriting task that requires integrating cognitive domains found deficient among people with PD-MCI. Based on evidence of visuospatial, working memory, organization, and psychomotor speed deficits among people with PD-MCI (e.g., [33,34,35]), we requested participants to fill in a check-formatted paper.

This study aims to further analyze the impact of the self-reported CF feature on suspected PD-MCI identification. We compare perceived general functional ability (PGF) and digitized handwriting performance measures of PD participants with and without suspected MCI based on their CF features. Furthermore, correlations between participants’ PGF, BDI scores, and digitized handwriting measures are analyzed. Finally, we address the question of whether participants’ global functional abilities, BDI scores, and digitized handwriting measures may predict the CF feature.

## 2. Materials and Methods

### 2.1. Participants

The study included 78 participants recruited at the Sheba Movement Disorders Institute in Israel and diagnosed with PD by expert neurologists. Inclusion criteria were an age of 40 years or older, living in Israel for at least 10 years, being functionally independent, with ability to read and speak Hebrew, normal or corrected hearing, and vision abilities. Furthermore, participants had to score 18 or less on the BDI [9] and 23 or more on the MoCA [36,37,38].

Exclusion criteria for all participants were significant systemic diseases, such as cancer or congestive heart failure; any neurological or psychiatric condition or damage; or treatment with antipsychotic or anticholinergic medications.

The 78 patients in the sample met the MDS clinical diagnostic criteria for probable or possible PD [39], and were Hoehn and Yahr Stage 1 or 2 [40]. The disease duration at inclusion was up to 10 years. A movement disorders neurologist assessed PD-related symptoms and signs using the MDS-UPDRS [12].

The PD patients were divided into two groups based on their CF features. Group A (1 point or more) were PD patients with CF difficulties (*n* = 25) suspected for PD-MCI (s.PD-MCI). Group B (less than 1 point) were PD patients without CF difficulties (*n* = 53). For detailed descriptions of both groups’ demographic characteristics, see [4] and Section 3.1.

### 2.2. Measures

A medical evaluation was conducted and self-report questionnaires that focused on functional cognition were administered to all participants.

*Daily Living Questionnaire (DLQ)* [13]

Our previous study implemented Parts A and B of the DLQ [13], which evaluates functional cognition through 52 items scored on a 5-point Likert scale. In the current study, we used Part C, which captures the individual’s PGF. The Part C score is comprised of seven items that ask the respondents about their overall ability to function, perceived changes in roles and responsibilities, and the degree of satisfaction with what they need and want to do in their daily lives [41]. The scores range from 1 (excellent/satisfied/no change) to 5 (weak/disappointing/completely changed). Thus, higher scores indicate a greater decline. For each group, we calculated a mean score for each item and a total mean score for all seven items. Cronbach’s alpha for the DLQ Part C general score was 90 in the current study.

*Computerized Penmanship Evaluation Tool* [24]

The ComPET, an online computerized handwriting evaluation system, was used in this study to collect and analyze handwriting data. The ComPET contains two main parts: data collection, which is not language-dependent, and data analysis, programmed using MATLAB software. Participants performed the writing task on A4-size lined paper affixed to the surface of a WACOM Intuos 4 digitizing tablet using a wireless electronic inking pen (Model GP-110). The displacement, pressure, and pen-tip angle were sampled at 100 Hz via a 1300 MHz Pentium (R) M laptop computer. The computerized system enables the collection of spatial, temporal, and pressure data while the subject is writing. Pen stroke on paper was measured each time the pen contacted the writing surface with an applied pressure above 50 non-scaled units. The ComPET also enables detecting pen motion in the air when the pen is up to 6 mm and pen-stroke time continues when the pen again contacts the paper. Pen-stroke height and width also are computed when the pen is on the paper. Notably, these measures relate to pen strokes and not letters because forming a letter may require combining two or more pen strokes.

**Figure 1:**
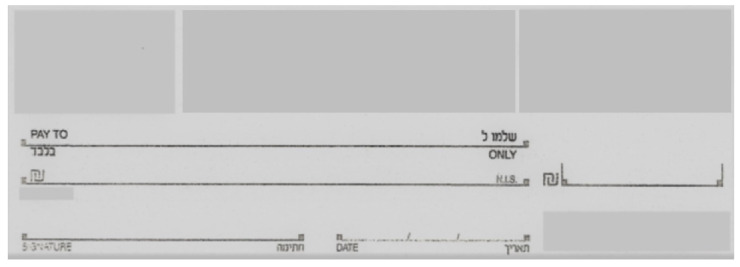
The fill-in-a-check task. The illustration shows the check format participants were required to fill in.

Participants were asked to fill in a check affixed to the digitizing tablet (Figure 1). They were provided the following written instructions to guide them: “Please fill in the check according to the details presented on this page. Pay to ‘self’, the amount of 5014 NIS, dated 30 March 2012, and sign your name in the designated place”. The computerized system gathered measures of the width and height of their pen strokes and performance time.

### 2.3. Procedure

The results presented in this study are part of the results of a larger study. Patients at the Sheba Movement Disorders Institute attended study visits (one or two visits no more than 2 weeks apart). During each visit, a movement disorder neurologist evaluated the patients according to both the Hoehn and Yahr Scale [40], and the MDS-UPDRS [12] after the patients took their dopaminergic medications (i.e., “on medication” state). We collected demographic, PD-related clinical, medical comorbidity, and PD-medication data from patient files and calculated total daily Levodopa equivalent dosages for each patient [42]. Additionally, a certified occupational therapist administered a neuropsychological battery, the self-report questionnaires focusing on participants’ daily functional abilities, and the handwriting test via the computerized system.

### 2.4. Statistical Analysis

We used IBM SPSS (version 23) for the analyses and tested statistical assumptions with the statistical significance of *p* = 0.05. To assess significant between-group differences in medical status and demographics, we used *t*-tests for continuous variables and Chi-squared tests for categorical variables. Given the significant between-group age difference and the correlations between the main study variables and age that indicated no significant correlation, we did not include age as a covariate variable in comparisons of the study’s main variables (PGF and handwriting measures). A Cronbach’s alpha analysis was implemented to check the internal variability of the PGF self-report (Part C of the DLQ). Tests of normality for each main study variable indicated normal distribution. Further comparison analysis at the mean-score and item levels of the PGF provided insight about which functional items best differentiated between the PD patients with and without suspected PD-MCI. We conducted additional *t*-tests to compare the handwriting process measures of both groups. Pearson correlations were conducted on the entire sample of PD patients (*N* = 78) between the handwriting process measure that differentiated the groups, the BDI score, the PGF score, and the CF feature. Finally, stepwise regression was conducted to examine whether the handwriting process measure that differentiated between the groups, the BDI score, and/or the PGF score may predict the CF feature above age.

## 3. Results

### 3.1. Participants’ Demographic and Medical Status

There were no significant differences between PD participants without CF difficulties (PD-n.MCI) and those suspected for MCI (s.PD-MCI) based on their CF feature in gender frequency (68% and 69.80% male) or in years of education (*M* = 16.4 years and *SD* = 3.68; *M* = 15.64 and *SD* = 3.67). No significant group differences were found for the percentile of participants treated with Levodopa (49.10% and 48%) or for PD duration since diagnosis in years (*M* = 4.21 and *SD* = 3.42, and *M* = 4.09 and *SD* = 2.88). The Hoehn and Yahr stage of the participants in the PD group ranged from 1.0 to 3.0, with the same median of 2.0 s, and of the participants in the PD-MCI group ranged from 1.0 to 2.5, with a median of 2.0. However, significant group differences were found for age ((*M* = 60.24 years and *SD* = 8.36; *M* = 66.26 and *SD* = 7.24), *t*(76) = −3.46, *p* = 0.004)); MoCA scores ((*M* = 26 and *SD* = 2.00; *M* = 24.98 and *SD* = 2.00), *t*(76) = 2.05, *p* = 0.043)); and BDI scores ((*M* = 7.79 and *SD* = 4.92; *M* = 5.43 and *SD* = 4.43), *t*(76) = 2.64, *p* = 0.010)).

In addition, Pearson correlations indicated no significant correlations between age and the main study variables (i.e., general functional DLQ score or handwriting measures). Thus, age was not held as a covariate in the between-group comparison.

### 3.2. Comparison of PGF Ability

Table 1 shows the significant group differences found for the participants’ PGF ability mean scores. The mean scores for each questionnaire also are presented to indicate the sources of the differences; high scores indicate more difficulties, less satisfaction, or greater change due to the disease. Although there were no significant differences for general thinking, we found significant differences for all other items capturing general functioning, roles changes, satisfaction level, and life change due to the disease.

### 3.3. Comparison of Objective Handwriting Measure (Fill in a Check)

As presented in Table 2, only the mean pen-stroke width significantly differentiated the groups, with the mean significantly smaller among participants with s.PD-MCI.

**Figure 2:**
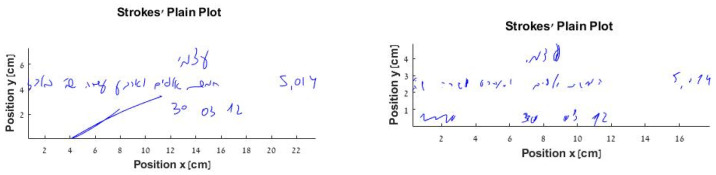
Examples of written fill-in-a-check task input by PD-n.MCI patient (**left**) and s.PD-MCI patient (**right**).

Two examples of the written fill-in-the-check task input are presented in Figure 2 to illustrate the differences in the mean pen-stroke width. The left input shows a mean pen-stroke width of 0.26 for a PD-n.MCI participant, whereas the right input represents a mean pen-stroke width of 0.10 for an s.PD-MCI patient.

### 3.4. Correlations between Handwriting Process Measure, PGF Ability, and BDI and CF Score: All Samples

Table 3 shows significant medium correlations between the PGF score, pen-stroke width, and CF feature. A significant low correlation was found between the BDI and CF scores. Interestingly, a medium significant correlation was found between the BDI and the PGF scores.

### 3.5. Prediction of CF Feature by Handwriting Process Measures, PGF Ability, and BDI Scores above Age

Finally, Table 4 presents the results of the stepwise regression conducted to find whether PGF ability, BDI, and handwriting performance measures predicted the CF feature. The results indicated that age accounted for 12% of the variance of the CF feature, i.e., *F*(1,72) = 11.08, *p* = 0.001; the PGF ability score added 20% to the prediction, i.e., *F*(2,72) = 17.95, *p* < 0.001; and the pen-stroke width added 8%, i.e., *F*(3,72) = 17.41, *p* < 0.001. As a whole, both the PGF ability score and the mean stroke width accounted for 28% of the variance of the CF feature above age. The BDI score did not contribute to the CF feature’s prediction and is not shown in Table 4.

## 4. Discussion

The aim of this study was to examine whether PGF ability and activity performance features of PD patients defined as suspected for MCI based on their self-reported CF feature would differ among this group and to find correlations between those measures and their CF feature prediction.

Results of the PGF questionnaire (DLQ Part C) subscale provide insight into the daily function-related feelings of the s.PD-MCI group compared to the PD-n. MCI group. As a whole, the final mean score of the s.PD-MCI group is significantly higher, indicating their lower functional ability, satisfaction, and role participation than that of the PD-n.MCI group.

The item-level scores do not indicate a decline in the general thinking ability of the s.PD-MCI group. However, that group’s functional ability is significantly different from the PD-n.MCI group, with all the items presenting higher mean scores. Specifically, people with s.PD-MCI reported substantially less satisfaction from doing what they need and want to do, and greater changes in their housekeeping, community, work roles, and lives due to the disease than did PD patients who are not suspected for MCI.

The significant difference found for mean pen-stroke width obtained using ComPET may shed further light on these patients’ daily functioning characteristics. Two previous studies found pen-stroke width to be a measure that significantly distinguished between participants with and without MDD who performed drawing and handwriting tasks on a computerized system [28,43]. In both studies, participants with MDD had significantly smaller pen-stroke width in the clock-drawing task [43] and in writing their names and surnames [28] than did those of the control group. The pen-stroke width alongside other digitized spatial measures, such as pen-stroke height and length, pen azimuth, and pressure measures, distinguished 89% of the MDD group in each study and more than 80% of all samples collectively.

The findings of the significantly smaller pen-stroke width among participants with certain emotional decline (MDD) were similar to the current results. Thus, it seems that the smaller mean pen-stroke width in the current study may reflect declining emotional health.

When considering the PGF questionnaire results and the decreased mean stroke width together, an interesting point arises. Previous studies reported correlations between handwriting measures and patients’ status/report among participants with varied pathologies (e.g., depression [28], Alzheimer’s disease [44], and developmental coordination disorders [31]). Given those results, we assume that an individual’s pen motions on the paper may reflect their motions in their real-life space. It seems that due to the cognitive decline (exhibited by their CF report) and accompanying emotional consequences, their space is decreasing. They do less and both their role and life space are diminished. The significant medium correlations found between the CF feature, PGF score, mean pen-stroke width, and BDI score enhance the message of this study’s results regarding the relationships between cognitive and emotional components. Such functional cognitive–emotional decline seems to lead people with suspected MCI to decrease their activities and participation, and thus their emotional and perhaps physical health and well-being, and vice versa.

As for the regression results, it is interesting to note that in a previous study, the DLQ and TOPS accounted for up to 35% of the variance. In contrast, the PGF and pen-stroke width predicted only 28% of the variance of the CF feature in this study. Such results support the need to enable participants to provide a detailed description of their daily function capabilities in the real world through appropriate questions based on theoretical and clinical knowledge. These descriptions may assist in capturing this hidden phenomenon of being at risk for MCI because referring to patients’ general functional abilities may not be accurate enough to provide a complete picture.

The regression results indicating that both the PGF ability feature and the mean stroke width accounted for 28% of the variance of the CF feature above age enhance support for the relationships found between pen-stroke width and the individual’s general functioning. It further supports the CF benefit as a possible assistance marker for suspected PD-MCI identification.

The study had some limitations. The sample was a small convenience sample that did not necessarily represent the typical population with PD. Further studies about the reliability and validity of the scale used in the current study to measure PGF abilities (i.e., DLQ Part C) are still needed despite the high reliability found in this study.

## 5. Conclusions

Future integration of varied analysis methods for the handwriting data taken from handwriting recognition methods [45], machine learning algorithms [46], and combing intelligent pen devices [47] may support the PD-MCI early detection process.

Thus, it appears that the self-reported CF feature might enable people to reflect their CF decline through this simple measure, which combines one cognitive item and six items related to ADL performance. Those items currently appear under the UPDRS *motor* experiences of the daily living subscale. However, participating in ADL also requires *cognitive* components such as inhibition (ignoring irrelevant stimuli not appropriate to the purpose), attention, working memory (action sequence), organization (what first, what next), spatial orientation (from which direction to begin dressing), visuospatial abilities (such as finding a car in a parking lot), and additional executive functions [48,49].

Given the associations between specific cognitive abilities and deficient ADL abilities [48], reporting through the CF feature even a slight decline in cognitive and ADL abilities—which reflects a decrease in functional cognitive abilities—may be self-reported support for PD-MCI identification.

Appreciable online, computerized, and cloud-based methods for cognitive evaluation have been previously reported (e.g., [50,51]). However, these developments rely on participants performing tasks only on the computer screen or self-reporting directly related cognitive abilities. Furthermore, these developments have not been studied specifically on people with PD. The uniqueness of our study is the method of evaluating functional cognition in real-life scenarios accompanied by objective measures of real-life performance, such as using a handwriting task that requires cognitive abilities.

## Figures and Tables

**Table 1:** Comparison of the item and mean final scores of the perceived general functional ability of s.PD-MCI and PD-n.MCI patients.

Variable	*M* (*SD*)			
	s.PD-MCI (*n* = 25)	PD-n.MCI (*n* = 53)	*t*(76)	*p*
1. General thinking	2.32 (0.99)	2.21 (0.85)	0.40	0.620
2. General functioning	3.12 (1.05)	2.63 (0.95)	2.02	0.046
3. Role change: housekeeping	3.20 (1.15)	2.13 (1.13)	3.83	<0.001
4. Role change: community or work	3.08 (1.17)	2.10 (1.24)	3.27	0.002
5. Satisfaction: doing things you need to do	2.80 (1.12)	2.06 (0.89)	3.14	0.002
6. Satisfaction: doing things you want to do	3.20 (1.19)	2.10 (0.89)	4.11	<0.001
7. Change in your life due to the illness	3.64 (0.86)	2.62 (0.91)	4.70	<0.001
Final mean score	3.04 (0.78)	2.26 (0.75)	4.23	<0.001

Legend: s.PD-MCI = patients suspected of Parkinson’s disease (PD) with mild cognitive impairment (MCI); PD-n.MCI = patients with PD but not MCI; *M* = mean; and *SD* = standard deviation.

**Table 2:** Comparison of the objective measures of the handwriting fill-in-a-check task of s.PD-MCI and PD-n.MCI patients.

Variable	*M* (*SD*)			
	s.PD-MCI (*n* = 25)	PD-n.MCI (*n* = 53)	*t*(76)	*p*
Performance time	44.130 (13.870)	44.920 (14.000)	−0.226	0.822
Mean pen-stroke width	0.164 (0.050)	0.054 (0.013)	−3.190	0.002
Mean pen-stroke height	0.285 (0.077)	0.307 (0.066)	−1.250	0.214

**Table 3:** Correlations between handwriting process measures, PGF ability, and BDI and CF feature: all samples.

	CF Feature	SWGM	BDI	PGF
CF feature		−0.428 **	0.382 **	0.530 **
SWGM	−0.428 **		−0.115	−0.173
BDI	0.382 **	−0.115		0.502 **
PGF	0.530 **	−0.173	0.502 **	

Legend: CF feature = cognitive functional feature; SWGM = mean pen-stroke width; BDI = Beck Depression Inventory; and PGF = perceived general functional ability. ** *p* < 0.01.

**Table 4:** Stepwise regression of the CF feature prediction by handwriting process measures, PGF ability, and BDI scores above age.

Variable	Model 1			Model 2			Model 3		
	B	SE B	β	B	SE B	β	B	SE B	β
Age	−0.024	0.007	−0.368 ***	−0.015	0.007	−0.229 *	−0.010	0.006	−0.157
PGF				0.292	0.063	0.473 ***	0.271	0.059	0.439 ***
SWGM							−0.230	0.690	−0.315 ***
*R* ^2^	0.13			0.33			0.43		
F	11.080 ***			17.95 ***			17.41 ***		

Legend: B = unstandardized regression coefficient; SE B = standard error for B; and β = standardized regression coefficient. * *p* < 0.05, *** *p* ≤ 0.001.

## Data Availability

The data presented in this study are available upon request from the corresponding author. The data are not publicly available due to the privacy of the participants.
